# A randomized controlled trial to reduce sedentary time in rheumatoid arthritis: protocol and rationale of the Take a STAND for Health study

**DOI:** 10.1186/s13063-020-4104-y

**Published:** 2020-02-12

**Authors:** Ana Jessica Pinto, Tiago Peçanha, Kamila Meireles, Fabiana Braga Benatti, Karina Bonfiglioli, Ana Lúcia de Sá Pinto, Fernanda Rodrigues Lima, Rosa Maria Rodrigues Pereira, Maria Claudia Costa Irigoyen, James Edward Turner, John P. Kirwan, Neville Owen, David W. Dunstan, Hamilton Roschel, Bruno Gualano

**Affiliations:** 1 Applied Physiology and Nutrition Research Group; Laboratory of Assessment and Conditioning in Rheumatology; Hospital das Clínicas HCFMUSP, Faculdade de Medicina FMUSP, Universidade de Sao Paulo, Av. Dr. Arnaldo, 455, 3° andar, São Paulo, SP 01246-903 Brazil; 2 Rheumatology Division, Faculdade de Medicina FMUSP, Universidade de São Paulo, Av. Dr. Arnaldo, 455, Sao Paulo, SP 05403-900 Brazil; 3 School of Applied Sciences, State University of Campinas, R. Pedro Zaccaria, 1300, Limeira, SP 13484-350 Brazil; 4 Heart Institute, Faculty of Medicine, University of Sao Paulo, Av. Dr. Enéas Carvalho de Aguiar, 44, São Paulo, SP 01246-903 Brazil; 5 Department for Health, University of Bath, Claverton Down Road, Bath, BA2 7AY UK; 6 Pennington Biomedical Research Center, 6400 Perkins Road, Baton Rouge, LA 70808 USA; 7 Baker Heart and Diabetes Institute, Melbourne VIC, Australia – 99 Commercial Road, Melbourne, Victoria 3004 Australia; 8 Centre for Urban Transitions, Swinburne University of Technology, John St, Melbourne, Victoria 3122 Australia; 9 Mary MacKillop Institute for Health Research, Australian Catholic University, 215 Spring St, Melbourne, Victoria 3000 Australia

**Keywords:** Sitting, Light-intensity physical activity, Rheumatic arthritis

## Abstract

**Background:**

Patients with rheumatoid arthritis spend most of their daily hours in sedentary behavior (sitting), a predisposing factor to poor health-related outcomes and all-cause mortality. Interventions focused on reducing sedentary time could be of novel therapeutic relevance. However, studies addressing this topic remain scarce. We aim to investigate the feasibility and efficacy of a newly developed intervention focused on reducing sedentary time, and potential clinical, physiological, metabolic and molecular effects in rheumatoid arthritis.

**Methods:**

The Take a STAND for Health study is a 4-month, parallel-group, randomized controlled trial, in which postmenopausal patients with rheumatoid arthritis will set individually tailored, progressive goals to replace their sedentary time with standing and light-intensity activities. Patients will be recruited from the Clinical Hospital (School of Medicine, University of Sao Paulo) and will be assessed at baseline and after a 4-month follow up. Outcomes will include objectively measured sedentary behavior (primary outcome) and physical activity levels, clinical parameters, anthropometric parameters and body composition; aerobic fitness, muscle function, blood pressure, cardiovascular autonomic function, vascular function and structure, health-related quality of life, and food intake. Blood and muscle samples will be collected for assessing potential mechanisms, through targeted and non-targeted approaches.

**Discussion:**

Findings will be of scientific and clinical relevance with the potential to inform new prescriptions focused on reducing sedentary behavior, a modifiable risk factor that thus far has been overlooked in patients with rheumatoid arthritis.

**Trial registration:**

ClinicalTrials.gov, NCT03186924. Registered on 14 June 2017.

## Introduction

Rheumatoid arthritis is an autoimmune disease characterized by chronic inflammation, joint damage, pain, fatigue, and physical disability [1]. Patients with rheumatoid arthritis have a greater risk of cardiovascular disease and premature mortality, which are partially explained by the complex interplay between chronic inflammation, adverse effects of drugs, associated comorbidities (e.g., dyslipidemias, insulin resistance, hypertension), and lifestyle [2–4]. Physical inactivity and sedentary behavior are modifiable risk factors that can aggravate disease symptoms and contribute to poor health outcomes [5].

The role of physical activity in the management of rheumatoid arthritis has dramatically shifted. In the 1970’s, bed rest and immobilization were recommended [6]. Now, it is well-known that exercise training improves disease symptoms, cardiometabolic risk factors, and physical fitness, which together may confer protection against premature mortality [7–9]. However, participation in higher-intensity physical activity may not be suitable for patients with rheumatoid arthritis, especially those with disabilities and active disease, conditions that may restrict moderate-to-vigorous physical activity. Light-intensity physical activity has been recently associated with lower cardiovascular risk, disability, and disease activity in rheumatoid arthritis [10]. Thus, interventions focused on replacing sedentary time with light-intensity physical activity could be of high clinical relevance.

Sedentary behavior (sitting) is strongly associated with poor health outcomes (e.g., cardiovascular disease, type 2 diabetes mellitus, some cancers) and all-cause mortality [11, 12]. Controlled laboratory studies have shown that active breaks in sedentary time (e.g., 2-min light-walking breaks every 20 min) for 5–8 h can improve cardiometabolic risk factors (e.g., reduced postprandial glucose and insulin) in general and clinical populations [13, 14]. Intervention studies focused on reducing sedentary time have also shown improvements in insulin sensitivity, lipid profile, body composition, and blood pressure in the general population and in the obese [15–17]. However, the impact of reducing sedentary time in rheumatoid arthritis remains underexplored.

Patients with rheumatoid arthritis spend 10.3 h/day on average in sedentary behavior [10, 18–21], which exceeds that in the general population (~ 7.5 h) [22], but is comparable to that in other clinical conditions (~ 9.4 h; pooled data for cardiovascular diseases [23], type 2 diabetes mellitus [24, 25]), and obesity [26, 27]). In these latter conditions, sedentary behavior is consistently related to morbidity and mortality [28–30]. Despite the paucity of evidence, it is plausible to assume that this might also hold true for patients with rheumatoid arthritis, since they commonly have cardiometabolic risk factors that can be aggravated by sedentary behavior [2–4]. To our knowledge, only a single study has addressed this topic, showing that an intervention involving general motivational counseling and text-message reminders resulted in reduced sedentary time (1.6 h/day), pain, and fatigue and improved quality of life in a Scandinavian cohort of patients with rheumatoid arthritis [31]. The cross-cultural validation of this finding in a Latin-American cohort with a lower socioeconomic status is necessary. In addition, exploring the putative mechanisms underlying the effects of reducing sedentary time requires novel studies. We aim to investigate the effects of a newly developed intervention focused on reducing sedentary time and its clinical, physiological, metabolic, and molecular effects in patients with rheumatoid arthritis.

## Materials and methods

### Objectives and hypotheses

We will test the feasibility and efficacy of a newly developed personalized intervention focused on replacing sedentary time with light-intensity physical activity in patients with rheumatoid arthritis. A multitude of techniques will be applied to evaluate the effects of the intervention on several outcomes, including sedentary time (primary outcome), physical activity levels, clinical parameters, cardiometabolic risk factors, and health-related quality of life. In addition, molecular analyses will be conducted in skeletal muscle and blood samples to gather knowledge on the potential underlying mechanisms by using targeted and non-targeted omic approaches. Finally, responders and non-responders (based on the reduction of sedentary time) will be compared to test the efficacy of reducing sedentary behavior on health-related outcomes in rheumatoid arthritis.

Our two main hypotheses are that (1) the intervention will be effective in reducing sedentary time, and (2) reducing sedentary time will improve clinical parameters, cardiometabolic risk factors, and health-related quality of life.

### Experimental design

We will conduct a 4-month, parallel-group, randomized controlled trial, in which patients will be assessed at baseline (PRE) and after 4 months (POST) for sedentary behavior (primary outcome) and physical activity levels; clinical parameters; anthropometric parameters and body composition; aerobic fitness; muscle function; blood pressure; cardiovascular autonomic function; vascular function and structure; health-related quality of life; blood samples and oral glucose tolerance test; immune function; muscle biopsy; and food intake. Sedentary behavior and physical activity levels will be also evaluated at the second month to check adherence to the intervention.

After baseline assessments, patients will be randomly allocated to either a control or intervention group using a simple randomization (1:1 ratio) procedure, by computer-generated random numbers in SAS 9.3 (SAS Institute Inc., Cary, NC, USA) for Windows. An external researcher will generate the allocation sequence and be contacted after patients’ enrollment. All assessors will be blinded to patients’ allocation and will be responsible for patients’ recruitment and enrollment. However, a trained researcher who will not be blinded to patients’ allocation will be responsible for assigning patients, and for applying and monitoring the Take a STAND for Health intervention. The control group will receive standard care, including general advice on healthy lifestyle. The intervention group will receive standard care supplemented with a specific personalized intervention aimed at reducing sedentary time (called Take a STAND for Health), which will not require alteration to usual care pathways (including use of any medication). In brief, this intervention comprises individually tailored progressive goals aimed at reducing sedentary time, which will be selected with the active participation of the patient. A trained researcher will assess adherence to the goals via phone calls and personal interviews throughout the follow up.

**Fig. 1:**
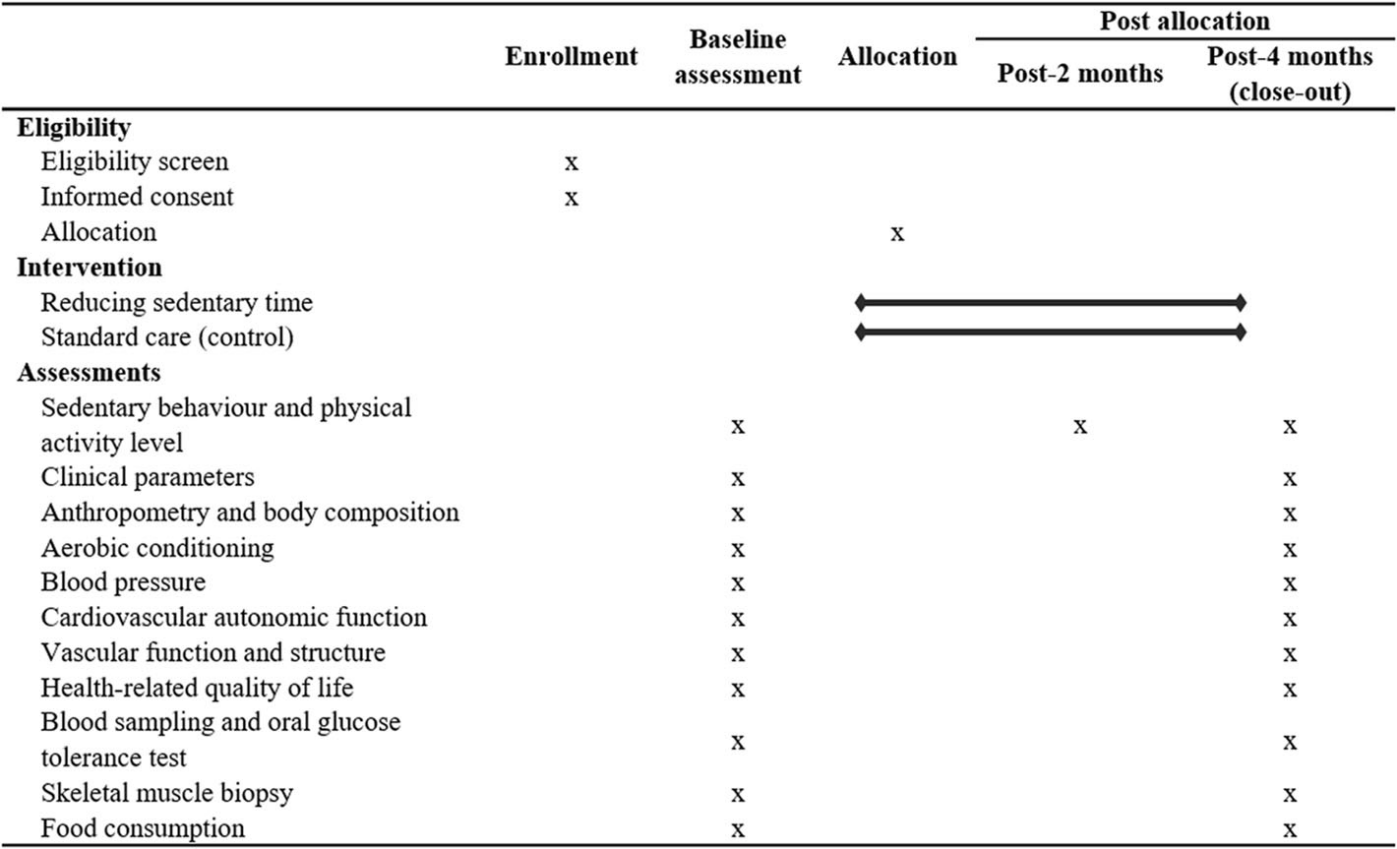
Overall schedule of enrollment, intervention, and assessments following the Standard Protocol Items: Recommendations for Interventional Trials (SPIRIT) checklist

**Fig. 2:**
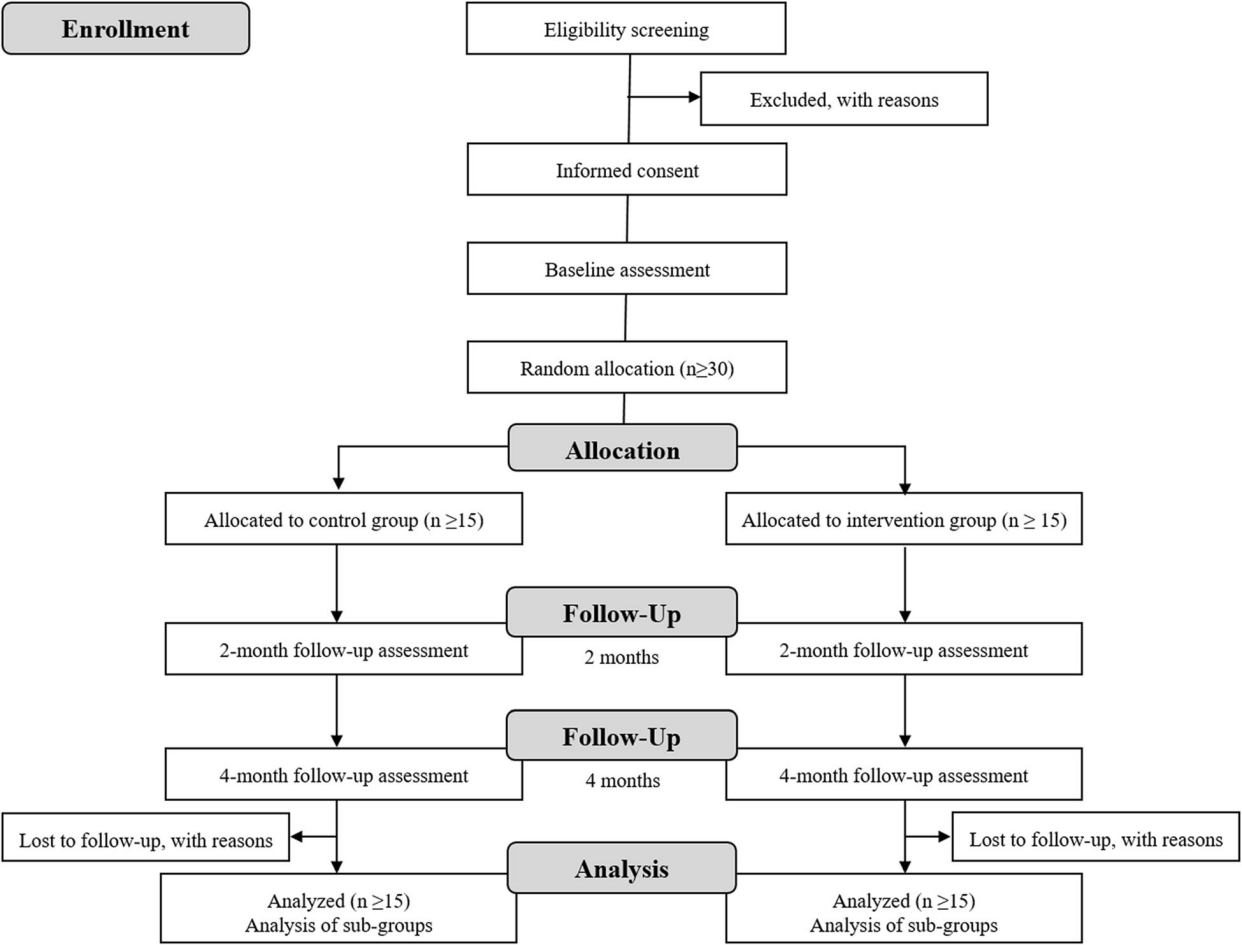
Consolidated Standards of Reporting Trials (CONSORT) flow diagram

The current study is registered in an international database of clinical research studies (clinicaltrials.gov, NCT03186924). This manuscript is described according to the Standard Protocol Items: Recommendations for Interventional Trials (SPIRIT) checklist (Fig. 1, Additional file 1) and the findings from this study will be reported according to the recommendations of the Consolidated Standards of Reporting Trials (CONSORT) guidelines (Fig. 2).

### Patient recruitment and selection

Patient recruitment and selection will take place at the Clinical Hospital (School of Medicine, University of Sao Paulo). Postmenopausal patients diagnosed with rheumatoid arthritis (*N* = 30) [32] will be recruited directly from the Rheumatoid Arthritis Outpatient Clinic of the Rheumatology Division. The exclusion criteria include (1) participation in structured exercise training programs within the last 12 months; (2) unstable drug therapy in the last 3 months prior to and during the study; (3) Health Assessment Questionnaire score > 2.0 (i.e., severe physical impairment).

#### Sample size

Sample size calculations were performed using G-Power® software, v. 3.1 (Universität Düsseldorf, Düsseldorf, Germany), based on the study by Lewis et al. that reported the effects of reducing sedentary behavior in an elderly population (i.e., a reduction of 52 min in total sitting time) [33]. According to the estimation, 24 patients (12 per arm) are required to achieve 95% power (α), with a significance level of 5% (β), and assuming an effect size of 0.58 for the primary outcome (i.e., sedentary time). Estimating a dropout rate of ~ 25%, at least 30 patients will be recruited. Considering that this sample size could be underpowered for some secondary outcomes, we will try to increase this estimated sample based on the feasibilities of our laboratory (including funding, capacity of research staff and facilities, and available patients), in line with contemporary recommendations [34, 35].

#### Ethical compliance

This trial has been approved by the local Ethical Committee (Commission for Analysis of Research Projects, CAPPesq; approval: 1.735.096). Patients will be required to sign an informed consent form before participation and all the procedures will be conducted in accordance with the Declaration of Helsinki revised in 2008. Patients will provide formal consent to share their data and samples, when applied, with the international research centers that cooperate in this study, in accordance with standard ethical procedures. In the case of ancillary studies, patients will be contacted to provide additional consent for the research team to use their data and biological specimens.

There will be no formal stopping rules, as this trial has minimum (if any) risk to the patients, given the characteristics of the intervention (very light physical activities). In addition, the follow up is relatively short, hampering any interim analysis that could precisely inform any eventual decision of interruption due to the lack of benefits or deleterious effects.

The researchers responsible for conducting the intervention and collecting the data along with at least two senior researchers will meet on a weekly basis, to discuss the progress of the protocol and eventual deviations from the original work plan. The ethics committee will be informed of any deviation. Upon study completion, the researchers will send a final report to the ethics committee, which will assess compliance to the ethics procedures.

### The Take a STAND for Health intervention

**Fig. 3:**
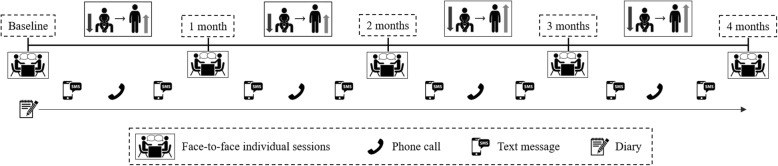
Overall design of the Take a STAND for Health intervention

The Take a STAND for Health program is a newly developed, goal-setting, behavioral intervention aimed at reducing sedentary behavior (Fig. 3). Overall, this intervention consists of five face-to-face individual sessions, lasting approximately 15–30 min each. A trained researcher will conduct the individual sessions and patients will be instructed to choose goals to reduce sedentary behavior in the following domains: transport, work, and/or leisure and social activities. Patients will receive supportive phone calls and/or text messages on a weekly basis to check adherence to the goals. In addition, compliance will also be verified during individual meetings. More details on the interventions are presented in the next sub-sections. In a small pilot study in healthy young patients, we found that this program reduced sedentary time by 38 min/day after just 2 weeks (for further details see “Results and lessons from the pilot study”).

#### The constructs of Take a STAND for Health 

This intervention is founded on the constructs of self-determination theory. This theory argues that people have inherent growth tendencies and innate psychological needs (i.e., autonomy, competence, and relatedness), which are the basis for intrinsic motivation and behavior. A positive environment (i.e., one that satisfies all of these needs) could result in increased motivation and enduring behavioral changes. Take a STAND for Health effectively incorporates all of these needs: autonomy is stimulated because patients actively take part in goal setting; competence is accomplished since the goals are individualized and achievable; and relatedness is fostered by the monthly individual meetings, and the integrating environment that is fostered by the supportive phone calls and text messages. The Take a STAND for Health intervention was based on the Small Steps program [33], which was developed on the same principles, and was shown to be effective in reducing sedentary time (by 51 min/day) in older adults.

#### Goals domains and description

Goals will be separated into the following domains: (1) transport, which involves reducing sedentary time during transportation (e.g., park further away from your destination except when carrying a heavy weight, or alight from the bus a stop before or after your destination); (2) work, which involves reducing sedentary behavior at the workplace (e.g., stand up every 30 min while performing activities in a seated position or stand up during meetings and invite your colleagues to join you); and (3) leisure/social activities, which involve reducing time spent in sedentary behavior during leisure time (e.g., stand up during advert breaks when watching television or walk with your dog at least twice a week).

#### Sessions aims and structure

During session 1, a trained researcher will explain the details of the intervention (e.g., aim, duration, frequency, compliance) to the patient. Subsequently, they will be asked to talk about their daily routines and choose goals to reduce time spent in sedentary behavior, including goals from each domain, from a list of prespecified items. Goals will be explained in more detail if necessary and all possible questions will be clarified before patients set the goal. Patients will be encouraged to actively select their own goals; however, the researcher will assist them in opting for goals that could be feasible to incorporate into their daily routine. Patients will be advised to adhere to their goals throughout the intervention and will receive a summary of the goal-setting plan and will be requested to return individually tailored feedback (using a diary) in the next session.

During the following sessions, each patient will be asked about the execution of her goals and encouraged to report barriers and facilitators to achieving her goals. If a patient is adhering to the goals, she will be encouraged to maintain her routine. If not, the researcher will discuss ways of overcoming the reported barriers; if a barrier is considered to be unresolvable, the patient will be guided in selecting a new goal. The patient will deliver the diary and receive another one to be filled out and returned in the next session. Patients will be permanently encouraged to increase the reduction in sedentary time or increase the frequency of sit-to-stand transitions. In the last session, each patient will be asked about the feasibility of and adherence to the intervention, and barriers and facilitators for reducing sedentary behavior throughout the intervention.

#### Results and lessons from the pilot study

Seventeen young healthy participants (8 women and 9 men; age 26.4 ± 3.4 years; body mass index (BMI) 24.4 ± 3.0 kg/m^2^) were recruited to undertake a pilot study of the Take a STAND for Health intervention (as per the description above), with the exception being the total number of the goals, which were set up to 15 originally. Before the intervention, sedentary time was assessed during 7 days (baseline), and participants were instructed to follow their usual routine. Thereafter, participants undertook the Take a STAND for Health intervention during the following 14 days (Post). Sedentary behavior was monitored throughout the entire 21-day period using ActivPAL micro™ (PAL Technology, Glasgow, UK). Generalized linear mixed models were analyzed to test changes in sitting, standing, and stepping time, with time as a fixed factor, and patients as a random factor. Cohen’s *d* effect size (ES) was also calculated to determine change in sedentary time. The level of significance was set at *p* ≤ 0.050.

**Fig. 4:**
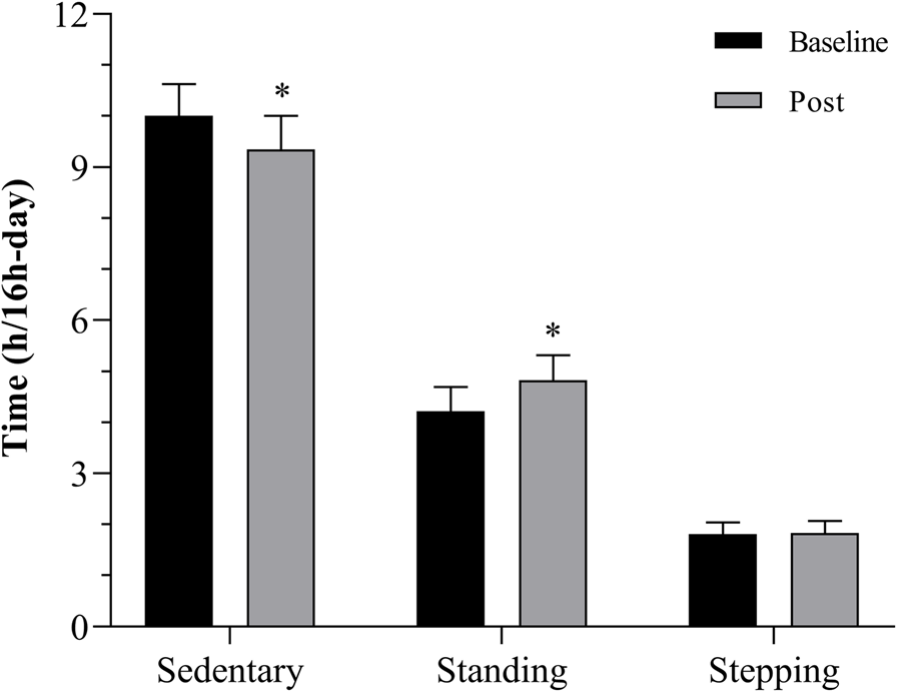
Time spent in sedentary behavior, standing, and stepping (hours/day) before and after the pilot study. *Significant difference when compared to baseline (*P* < 0.050)

Participants spent most daily hours in sedentary behavior (10.0 ± 1.3 h/day), followed by standing and stepping (4.2 ± 0.9 and 1.8 ± 0.5 h/day, respectively). After the intervention, participants reduced their time spent in sedentary behavior by 0.6 h/day (*p* = 0.032; 95% confidence intervals (CI) 0.1, 1.2 h/day; ES = 0.55), increased time spent standing (mean difference 0.6 h/day; 95% CI 0.2, 1.0; *p* = 0.006), and maintained time spent stepping (mean difference 0.02 h/day; 95% CI − 0.2, 0.2; *p* = 0.821) (Fig. 4). After the intervention, participants reported that it was difficult to remember and follow all selected goals. According to several participants, the number of goals was excessive, and occasionally they did not fit their routines. Based on this feedback, we decided to reduce the number of goals and maintain only those with the best chance of being effectively incorporated into the patients’ routines.

### Study procedures

#### Sedentary behavior (primary outcome)

Postural allocation (sitting, standing, and stepping) will be measured using activPAL micro™ (PAL Technology, Glasgow, UK) activity-based accelerometers. Patients will wear the accelerometer for 7 days consecutive (24 h/day); the accelerometer will be fitted using tape (3 M, Tegaderm®, adhesive tape) onto the right medial front thigh, orientated with the x-axis pointing downward, y-axis horizontally to the left and z-axis horizontally forward. Data will be exported from the device using ActivPAL3™ software, v. 7.2.32 (PAL Technology, Glasgow, UK). ActivPAL™. Data will be reported as follows: time spent sitting and lying (hours/day), in prolonged sitting (hours/day), standing (hours/day), and stepping (hours/day), number of breaks in sedentary time, and mean daily waking time (calculated as: 24 h − time spent sleeping). All data will be standardized to a 16-h day to avoid bias from differences in patients’ wear time, using the formula: (data × 16)/wear time.

#### Physical activity level

Physical activity levels will be objectively measured using the actiGraph GT3X® accelerometers (ActiGraph, Pensacola, FL, USA). All patients will be instructed to wear the accelerometer during waking hours for 7 days consecutive, except when bathing or swimming. The device will be worn on a belt at the waistline on the right side of the hip. Data will be exported in 60-s epochs using ActiLife 6 software, v. 6.11.9 (ActiGraph, Pensacola, FL, USA). Patients will have to accumulate at least 10 h of valid activity recordings per day for at least 4 days, including one weekend day. Non-wear periods will be defined as intervals of at least 60 min of zero activity counts, assuming as tolerance no more than 2 min of counts between 0 and 100. Freedson cut-points will be used to define epochs: sedentary time (< 100 counts per minute (cpm)), light-intensity physical activity (≥ 100 to < 1952 cpm), and moderate-to-vigorous physical activity (≥ 1952 cpm) [36]. Actigraph GT3X® data will be reported as follows: time spent in sedentary behavior (hours/day), light-intensity physical activity (hours/day), moderate-to-vigorous physical activity (minutes/day), and moderate-to-vigorous physical activity accrued in ≥ 10-min bouts (minutes/day), total counts, and mean daily wear time. All data will be standardized to a 16-h day to avoid bias from differences in patients’ wear time, using the formula: (data × 16)/wear time.

#### Clinical assessment

Disease activity will be assessed by the Disease Activity Score in 28 joints [37], which is based on the number of tender and swollen joints, C-reactive protein or erythrocyte sedimentation rate, and the patient’s general health status. Higher scores represent more severe disease activity. The Health Assessment Questionnaire [38], which evaluates physical functioning in eight domains of daily life, will also be used; higher scores represent greater disability. Disease duration, current dose of prednisone, current use of biological agents (e.g., anti-TNF, anti-IL6, anti-IL1, B cell depleting-agents, and T cell activation-inhibiters), non-biological disease-modifying anti-rheumatic drugs (e.g., methotrexate, leflunomide, and hydroxychloroquine), and other medications will be obtained by reviewing medical records and interviewing patients. Pain will be assessed using a visual analogic scale [39], in which patients grade their pain using a 10-point scale; 0 means no pain and 10 means severe or unbearable pain. Fatigue will be assessed by the Fatigue Severity Scale [40] in which lower scores indicate lower fatigue.

#### Anthropometry and body composition

Height will be measured by a wall-mounted stadiometer. Body mass will be measured by a digital scale, with a sensitivity of 100 g. BMI will be calculated using the following equation: body mass (kg)/height (m)^2^. Waist circumference will be measured using a plastic tape measure placed around the smallest circumference between the lowest margin of the ribs and the upper margin of the iliac crest, with subjects standing. Body composition (i.e., bone, lean and fat mass, and visceral adipose tissue) will be measured by dual-energy x-ray absorptiometry (DXA), using a Lunar iDXA densitometer (GE Healthcare, WI, USA). All assessments will be performed by the same trained technician.

#### Aerobic conditioning

Patients will undergo a maximal graded exercise test on a treadmill (Centurion 200, Micromed, Brazil), with increments in velocity and grade at every minute until volitional exhaustion. Oxygen consumption (VO_2_) and carbon dioxide output will be obtained through breath-by-breath sampling and expressed as a 30-s average using an indirect calorimetric system (Cortex - model Metalyzer IIIB, Leipzig, Germany). Heart rate (HR) will be continuously recorded at rest, during exercise, and at recovery, using a 12-lead electrocardiogram (Ergo PC Elite, Inc. Micromed, Brazil). The test will be considered maximal when one of the following criteria is met: plateau in heart rate and VO_2_ with incremental workloads, respiratory exchange ratio > 1.1, and HR no less than 10 beats below age-predicted maximal HR. VO_2_ peak will be considered as the average of the final 30 s of the test [41]. Ventilatory thresholds will be identified following previously described procedures [42]. In brief, the ventilatory anaerobic threshold will be determined when ventilatory equivalent (VE) for VO_2_ (VE/VO_2_) increases without a concomitant increase in ventilatory equivalent for carbon dioxide (VE/VCO_2_). The respiratory compensation point will be determined when VE/VO_2_ and VE/VCO_2_ increase simultaneously.

#### Muscle function

Muscle function will be evaluated by the Timed-Stands, the Timed-Up-and-Go, and handgrip tests. A familiarization trial will be performed at least 48 h prior to the tests.

The Timed-Stands test evaluates the maximum number of stand-ups that a subject can perform from a standard armless chair within 30 s [43]. The Timed-Up-and-Go test evaluates the time required for the subject to rise from a standard armless chair, walk towards a line drawn on the floor 3 m away, turn, return, and sit back down again [44]. Patients will perform two maximal attempts of each test, with 2-min recovery periods between sets. Subsequently, patients will perform the handgrip test using a handgrip dynamometer (Takei A5001 Hand Grip Dynamometer, Takei Scientific Instruments Co., Ltd., Tokyo, Japan). The protocol consists of three maximal isometric contractions during 5 s interspersed with 60-s recovery periods. Patients will be instructed to squeeze the dynamometer as hard as possible. The maximum score achieved will be recorded in kilograms [45].

#### Blood pressure

Blood pressure will be measured by the auscultatory technique using a non-mercury sphygmomanometer [46]. All measurements will be taken in the same arm by a trained evaluator.

A random sub-sample of patients will undertake a 24-h ambulatory monitoring of arterial pressure (Dyna-MAPA, CARDIOS, Sao Paulo, Brazil). The monitoring device will be programmed to take readings every 15 min. Patients will record their activities in a diary during a 24-h period, including when they slept and woke up. Data will be analyzed using the Dyna-MAPA software (only data with at least 80% of the reading will be considered) and will be reported as 24-h, daytime and night-time, mean systolic and diastolic blood pressure; 24-h, daytime and night-time, systolic and diastolic blood pressure load (calculated as percentage of values above 130/80, 135/85, and 120/70 mmHg); morning surge in systolic and diastolic blood pressure (calculated as follows: mean systolic/diastolic blood pressure for 2 h after wake up – the lowest 2-h mean values of systolic/diastolic blood pressure during sleep); and nocturnal systolic and diastolic blood pressure fall (calculated as follows: [(mean day time systolic/diastolic blood pressure – mean night time systolic/diastolic blood pressure)/mean day time systolic/diastolic blood pressure] × 100).

#### Cardiovascular autonomic function

A random sub-sample of patients will undertake cardiovascular autonomic assessments, which will comprise continuous assessments of heart rate using a 3-lead electrocardiogram, beat-by-beat blood pressure via finger photoplethysmography (Finometer, Finapress Medical System, Arnhem, The Netherlands), and muscle sympathetic nerve activity via microneurography. These signals will be registered using a data acquisition system (Powerlab, AD Instruments, São Paulo, Brazil) and Labchart software (AD Instruments, São Paulo, Brazil), with a sampling rate of 1000 Hz per channel. Assessments will be performed at rest in a supine position. Patients will be instructed to remain quiet and to breathe spontaneously during the 30-min assessment.

Heart rate variability will be analyzed in 5-min segments during rest using the CardioSeries software (v 2.4, São Paulo, Brasil) [47]. Initially, the RR interval time series will be generated from the electrocardiographic signals. Afterwards, the time domain parameters - standard deviation of RR intervals and square root of the mean’ of the sum of the squares of differences between adjacent normal RR interval - will be calculated. For frequency domain analysis, the RR interval time series will be detrended (smooth prior), resampled at 4 Hz, and decomposed using the fast Fourier transform algorithm. The components of low (LF, 0.04–0.15 Hz) and high (HF, 0.15–0.4 Hz) frequencies will be calculated as described elsewhere [48].

The analysis of muscle sympathetic nerve activity will be performed using Labchart (v 2.4, São Paulo, Brasil) software. The sympathetic bursts will be automatically identified using the automatic peak detection function. Occasional misdetections will be manually corrected by an experienced evaluator. The muscle sympathetic nerve activity will be expressed as burst frequency (bursts/minute) and burst incidence (burst/100 heart beats).

Cardiac and sympathetic spontaneous baroreflex sensitivity will be assessed from the fluctuations of the RR interval, blood pressure, and muscle sympathetic nerve activity, using CardioSeries (v. 2.4, São Paulo, Brasil) software. Cardiac baroreflex sensitivity will be assessed using the sequence technique [49]. Sympathetic baroreflex sensitivity will be assessed from the linear regression analysis between the area of sympathetic bursts and the corresponding diastolic blood pressure. The slope of the linear regression line will be used as an index of sympathetic baroreflex sensitivity [50].

#### Vascular function and structure

A random sub-sample of patients will undertake the vascular assessments. All vascular assessments will be performed in the supine position and by an experienced investigator blinded to the group allocation.

Carotid intima-media thickness will be assessed according to current guidelines [51]. Patients will remain with the head rotated to the left and a linear transducer (7–10 MHz) attached to a high-resolution ultrasound machine (GE Logiq E, GE Medical, Milwaukee, WI, USA) will be positioned perpendicularly to the right common carotid artery (i.e., longitudinal plane), 1–2 cm below the bifurcation. Ultrasound parameters will be modified to optimize the appearance of the intima border along the vessel. Measurements will be performed in three distinct angles and will be recorded for 30 s. The analysis of carotid intima-media thickness will be performed using edge detection and wall tracking software (Cardiovascular Suite, Quipu®, Pisa, Italy).

Flow-mediated dilation of the brachial and superficial femoral arteries will be assessed according to current guidelines [52]. For brachial flow-mediated dilation, patients will be positioned with their right arm extended at an angle of ~ 80° from the torso and immobilized with foam supports. A manual pneumatic cuff will be positioned at the forearm to provide the ischemic stimulus. A linear transducer (7–10 MHz) attached to a high-resolution ultrasound machine (GE Logiq E, GE Medical, Milwaukee, WI, USA) will be used to assess brachial artery diameter at the distal third of the upper right arm. For superficial femoral artery flow-mediated dilation analysis, patients will be positioned with their right thigh externally rotated, the cuff will be positioned 1–2 cm above the knee, and the ultrasound transducer will be placed on the distal thigh.

When a satisfactory image is acquired, the probe will be kept stable and the ultrasound parameters will be set to optimize the B-mode image of the lumen-arterial wall interface. Continuous Doppler blood flow velocity will also be analyzed using an insonation angle ≤ 60° and the sample volume will be placed in the middle of the artery. Initially, a 1-min baseline diameter and blood flow velocity recordings will be acquired and then the forearm cuff will be inflated (~ 200 mmHg) for 5 min. Recordings will be resumed 30 s before cuff deflation and continued for 3 min thereafter (5 min for the superficial femoral artery).

Offline analyses of diameters, blood flow, and shear rate will be performed using edge detection and wall tracking software (Cardiovascular Suite, Quipu®, Pisa, Italy). Flow-mediated dilation will be calculated as the percentage rise (peak − baseline) in brachial/superficial femoral diameter obtained after cuff release in relation to the preceding baseline diameter. Time to peak dilation, and baseline anterograde and retrograde shear rate will be calculated as described elsewhere [52].

#### Health-related quality of life

Health-related quality of life will be assessed by the SF-36 questionnaire [53], in which scales (physical function, role-physical, bodily pain, general health, vitality, social function, role-emotional) range from 0 to 100. Higher scores indicate better quality of life.

#### Blood sample processing and analysis

**Fig. 5:**
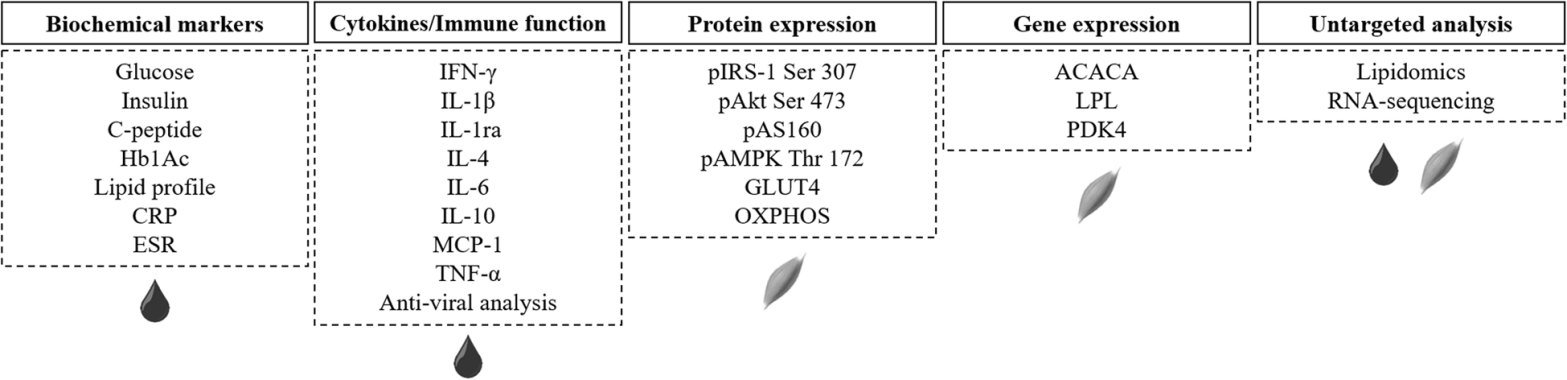
Summary of the blood and muscle analyses. ACACA, acetyl-coenzyme A carboxylase α; Akt, protein kinase B; AS160, Akt substrate of 160 kDa; AMPK, phosphorylated 5′ adenosine monophosphate-activated protein kinase; CRP, C-reactive protein; ESR, erythrocyte sedimentation rate; GLUT4, glucose transporter type 4; HbA1C, glycosylated hemoglobin; Lipid profile (triglycerides, total cholesterol, HDL, LDL, and VLDL); LPL, lipoprotein lipase; OXPHOS, oxidative phosphorylation complexes; PDK4, pyruvate dehydrogenase kinase 4

Blood samples (40 ml) will be collected after a 12-h overnight fast, for measuring the following: glucose, insulin, c-peptide, glycosylated hemoglobin (Hb_A1C_), lipid profile (i.e., high-density lipoprotein (HDL) cholesterol, low-density lipoprotein (LDL) cholesterol, very low-density lipoprotein (VLDL) cholesterol, total cholesterol, and triglycerides), C-reactive protein, erythrocyte sedimentation rate, and cytokines (i.e., IFN-γ, IL-1, IL-1ra, IL-4, IL-6, IL-10, monocyte chemoattractant protein 1 (MCP-1), and TNF-α). Blood samples will be collected in vacutainer tubes and subsequently analyzed at the Clinical Hospital Central Laboratory (School of Medicine, University of Sao Paulo). An aliquot will be centrifuged and stored at − 80 °C for analysis of cytokines and other molecular analyses. A summary of these analyses is provided in Fig. 5.

Glucose will be assessed using a colorimetric enzymatic assay (Bioclin, Belo Horizonte, Brazil). Insulin and peptide-C will be assessed using a radioimmunoassay technique (Diagnostic Products Corporation, Sao Paulo, Brazil). Total cholesterol, HDL, LDL, VLDL, and triglycerides will be assessed using enzymatic colorimetric assays (CELM, Sao Paulo, Brazil). C-reactive protein will be determined by immunoturbidimetry. Erythrocyte sedimentation rate will be assessed using an automated analyzer. Cytokines will be analyzed through a multiplex human panel (Billerica, MA, EMD Millipore, Milliplex®).

#### Oral glucose tolerance test

A 2-h oral glucose tolerance test will be performed at PRE and POST. Blood samples will be collected after a 12-h overnight fast, and at 30, 60, 90, and 120 min after ingestion of 75 g of glucose. Area under the curve (AUC) for glucose, insulin, and C-peptide, and Matsuda index, homeostatic model assessment-insulin resistance (HOMA-IR) and HOMA-B cell function (HOMA-B) will be calculated as surrogates of insulin resistance.

#### Lipidomics (non-targeted)

PRE and POST intervention serum samples (10 μL) will be collected from a random sub-sample of 20 patients (Fig. 5). Samples will be extracted in a single-phase extraction with 200 μL of CHCl3:MeOH (2:1) and 10 μL of an internal standard mix containing between 50 and 1000 pmol each of 23 non-physiological and stable isotope-labeled lipid standards. Sample analysis will be performed by electrospray ionization tandem mass spectrometry with the use of an Agilent 1200 liquid chromatography system combined with an Applied Biosystems API 4000 Q-TRAP mass spectrometer with a turbo-ion spray source (350 °C) and Analyst 1.5 data system. Solvent composition will comprise tetrahydrofuran-methanol-water with 10 mM ammonium format, at the following ratios; solvent A 20:20:60 and solvent B 75:20:5. All lipid species will be separated under gradient conditions at 300 μL/min, the gradient starting at 0% solvent B to 45% solvent B over the first minute, to 55% solvent B over 6 min, up to 80% solvent B over 1 min, up to 100% solvent B over 2 min, holding at 100% solvent B for 2 min, quickly back to 0% solvent B over 0.2 min and then holding at 0% solvent B until the next injection at 15.5 min. A total of 338 lipid species will be analyzed by multiple reaction monitoring experiments. This type of lipidomic measure is semi-quantitative, because stable isotope internal standards are not available for each individual lipid species. However, the precision of such measures is considered good (average coefficient of variation (CV) typically < 10%). We will report fasting lipid concentrations to provide an indication of the relative abundance of each lipid species or class. Lipid concentrations reported for lipid classes and subclasses will be calculated from the sum of the individual lipid species within each class. Finally, the percentage change in lipid species (PRE to POST) will be compared between groups (control and intervention groups).

#### Assessment of anti-viral T cell function

We will assess anti-viral T cell function as a measure of overall immune competency using Enzyme linked immunospot (ELISpot) technology in a random sub-sample of 20 patients (Fig. 5). For this analysis, an additional blood sample (25 ml) will be drawn into sodium heparin vacutainers. Blood will be diluted 1:1 with Roswell Park Memorial Institute (RPMI) medium, and layered on top of lymphocyte separation medium for isolation of peripheral blood mononuclear cells by density gradient centrifugation using standard methods. Peripheral blood mononuclear cells will be frozen slowly (− 1 °C per minute in a Nalgene Mr. Frosty freezing container) at − 80 °C, and after 12 h, stored in liquid nitrogen until assay. For the ELISpot assay, cells will be thawed rapidly to 37 °C, washed twice in RPMI (10% fetal calf serum, 1% penicillin and streptomycin) and rested for approximately 16 h in a humidified incubator (37 °C, 5% CO_2_). Cells will then be washed, counted, and added to polyvinylidene fluoride (PVDF) membrane plates (coated with anti-human IFN-γ antibodies) and stimulated for 16 h in separate conditions using immuno-dominant and conserved antigens from common viruses, including influenza, varicella zoster virus, Epstein Barr virus and cytomegalovirus. Stimulation will be via a human leukocyte antigen (HLA)-independent approach with overlapping peptides spanning the length of each antigen. Following stimulation, the ELISpot assay will be developed according to manufacturer instructions. These procedures will be undertaken in batches at the University of Sao Paulo, and stabilized assay plates will be shipped to the University of Bath in the UK for reading via an automated ELISpot plate reader (Autoimmun Diagnostika GmbH, Germany).

#### Food consumption

Food consumption will be assessed by means of three 24-h food recalls undertaken on separate days (i.e., 2 weekdays and 1 weekend day), using a visual aid photo album of real foods, which consists of listing all foods and beverages consumed during the prior 24 h. Patients will be instructed to maintain the same diet throughout the study. Energy (kilocalories) and macronutrient (grams and total percentage) intake will be calculated by a trained nutritionist using Dietbox® software (online version).

#### Mechanistic analyses

##### Skeletal muscle biopsy

Percutaneous muscle biopsies will be performed in a random sub-sample of 20 patients at PRE and POST, immediately after the oral glucose tolerance test. In brief, muscle biopsies will be obtained under local anesthesia (2–3 mL of 1% xylocaine) from the muscle vastus lateralis, using a 5-mm Allendale needle with suction. Immediately after the biopsy procedure, the sample will be blotted dry and trimmed of visible adipose and connective tissue using a standard dissecting microscope. Four portions of the specimen (~ 30–100 mg) will be snap frozen in liquid nitrogen and stored at − 80 °C.

##### Non-targeted analysis

We will perform RNA sequencing (RNA-seq; Fig. 5). Total RNA will be extracted, homogenized from ~ 20 mg of muscle tissue using TRIzol reagent (Invitrogen®), and isolated according to the RNeasy Fibrous Tissue Mini Kit (Qiagen®) protocol, using a Kinematica™ Polytron™ PT 1300 (FisherScientific®). Total RNA will be determined spectrophotometrically at 260 nm (GE Healthcare®) and RNA integrity number (RIN) will be checked by capillary electrophoresis using a Bionalyzer 2100 (Agilent®). For each sample, ~ 3 ul of total RNA extract will be delivered to the Pennington Biomedical Research Center Genomics Core for RNA sequencing (RNA-seq) analysis. Sample concentration will be normalized and complementary DNA (cDNA) pools will be created for each sample, and subsequently tagged with a barcoded oligo adapter to allow for sample-specific resolution. Sequencing will be carried out using an Illumina HiSeq 2500 platform (Illumina®) with a 50-bp single end reads. The quality of RNA-seq data will be checked using FastQC v0.10.0 (Barbraham Bioinformatics®). Alignment to the reference genome (rn5, UCSC), differential expression based on counts per million mapped reads (CPM), and post-analysis diagnostics will be carried out. RNA-seq data will be deposited in National Institutes of Health Gene Expression Omnibus (NIH GEO) and normalized CPM values for all measured genes will also be provided. To validate the RNA-seq data, gene and protein expression will be then determined by quantitative real-time PCR (qRT-PCR) and western blotting, as described in the following sections.

##### Targeted analyses

Gene expression will be determined by qRT-PCR (Fig. 5). In brief, total RNA isolation, quantification, and purity will be carried out as described above. Gene expression will be determined by qRT-PCR analyses using the microglobulin (*B2M*) gene as a housekeeping gene. All qRT-PCR reaction mixtures will be prepared using a Superscript Platinum One-Step kit (Invitrogen, CA, USA) with incorporated Maxima SYBR Green/ROX qPCR Master Mix (Applied Biosystems, CA, USA) on a Step One Thermocycler (Applied Biosystems, CA, USA). The messenger RNA (mRNA) levels of the genes lipoprotein lipase (*LPL*), pyruvate dehydrogenase kinase 4 (*PDK4*), and aetyl-CoA carboxylase alpha (*ACACA*) will be analyzed. Potential genes that emerge from the RNA-seq analysis will be validated using qRT-PCR analysis. Relative change in gene expression (Δ-ΔCq) will be calculated by subtraction of the ΔCq at PRE (used as a calibrator) to the corresponding ΔCq at POST. Finally, fold change will be determined as 2-Δ-ΔCq. All sense and reverse primers used for each gene will be reported along with the results.

Protein expression will be determined by western blot (Fig. 5). In brief, muscle samples will be homogenized in ice-cold lysis buffer. Equal loading of samples (25–40 μg) and transfer efficiency will be monitored with the use of 0.5% Ponceau S staining of the blot membrane. Primary antibodies involved in the insulin signaling pathway (phosphorylated insulin receptor substrate 1 (pIRS-1 Ser 307), phosphorylated protein kinase B (pAkt Ser 473), Akt substrate of 160 kDa (pAS160), phosphorylated 5′ adenosine monophosphate-activated protein kinase (pAMPK Thr 172), and total glucose transporter type 4 (GLUT4)) and oxidative capacity (mitochondrial complexes (OXPHOS)) will be incubated overnight at 4 °C. For each protein, binding of the primary antibody will be detected by peroxidase-conjugated secondary anti-rabbit or anti-mouse antibodies using chemiluminescence detected by ImageQuant LAS 4000 (GE Healthcare®), quantified by densitometry (Scion Image®), and normalized to respective total protein or housekeeping proteins. Potential candidates emerging from RNA-seq analysis will be validated using western blot analysis.

### Statistical analysis

Data normality will be tested using the Kolmogorov-Smirnov or the Shapiro-Wilk *W* test. Parametric data will be presented as mean ± 95% CI. Non-parametric data will be log-transformed and presented as back-transformed mean ± 95% CI. The ES (Cohen’s *d*) will be calculated and interpreted as small (0.2–0.4), medium (0.5–0.7), or large (≥ 0.8).

Generalized linear mixed model analyses will be performed for each dependent variable, with group and time as fixed factors and patients as a random factor; the models will be adjusted by age, BMI, disease activity, and if different between groups, baseline values of the outcome variable. In the case of significant *F* values, a post-hoc test with Tukey’s adjustment for multiple comparisons will be performed. Analyses will be conducted according to the intention-to-treat principle, in which missing values will be handled by the generalized linear mixed model by using maximum likelihood to estimate the parameters of the model. To test the influence of reducing sedentary time on health-related outcomes, patients will be allocated into “responder” and “non-responder” sub-groups (according to changes in sedentary time); thereafter, the dependent variables will be compared between them.

Data analysis will be performed using the SAS 9.3 (SAS Institute Inc., Cary, NC, USA) for Windows. The level of significance will be set at *p* ≤ 0.050.

## Discussion

Excessive time spent in sedentary behavior has been associated with poor health outcomes and all-cause mortality [11, 12]. Patients with rheumatoid arthritis spend most of their daily hours in sedentary behavior [10, 18–21]. Therefore, interventions aimed at reducing sedentary time have potential relevance to disease and cardiovascular risk management in these patients. The Take a STAND for Health study aims to comprehensively investigate the clinical, physiological, metabolic, and molecular effects of reducing sedentary behavior in rheumatoid arthritis.

The strengths of this study include the randomized controlled study design, which will allow us to investigate the effects of reducing sedentary time prospectively versus a control group; the use of objective measures of sedentary behavior and physical activity; the comprehensive clinical, physiological, and metabolic assessments, using robust techniques; the attempt of revealing new mechanisms, using both targeted and non-targeted approaches; and the evaluation of a novel, individually tailored intervention previously refined by a pilot study that has the potential of being delivered in real-world contexts.

This study could generate a novel body of evidence with the potential to advance knowledge on the clinical effects of reducing sedentary behavior and its underlying mechanisms in rheumatoid arthritis. Our results could inform evidence-based prescriptions focused on reducing sedentary time, which is a modifiable, overlooked risk factor in this disease.

### Trial status

Protocol version number: NCT03186924 (first version). Date of protocol registration, 14 June, 2017.

Recruitment began in December, 2017 and we expect to conclude patients’ recruitment by February 2020 and the 4-month follow-up assessments by June 2020.

## Supplementary information

**Additional file 1.** SPIRIT 2013 checklist: recommended items to address in a clinical trial protocol and related documents.

## Data Availability

The data generated and/or analyzed during the current study will be available in the intranet repository from the Clinical Hospital of the School of Medicine of University of Sao Paulo (Prontmed), which is password-protected and safely stores medical information from all patients of the Clinical Hospital. Final data produced by this study will be compiled as PDF documents in an electronic device (i.e., external hard drive (HD)) as well as in a cloud computing system, which will be appropriately password-protected. The datasets used and/or analyzed during the current study will be available from the corresponding author on reasonable request.

## Abbreviations

ACACA: Acetyl-coenzyme A carboxylase α
Akt: Protein kinase B
AMPK: Phosphorylated 5′ adenosine monophosphate-activated protein kinase
AS160: Akt substrate of 160 kDa
AUC: Area under the curve
BMI: Body mass index
bp: Base pairs
CI: Confidence interval
cpm: Counts per minute
CRP: C-reactive protein
ELISpot: Enzyme-Linked ImmunoSpot
ES: Effect size
ESR: Erythrocyte sedimentation rate
GLUT4: Glucose transporter type 4
HbA1C: Glycosylated hemoglobin
HR: Heart rate
IFN-γ: Interferon gamma
IL: Interleukin
IL-1ra: Interleukin 1 receptor antagonist
kDa: KiloDalton
LPL: Lipoprotein lipase
OXPHOS: Oxidative phosphorylation complexes
PDK4: Pyruvate dehydrogenase kinase 4
RNA-seq: RNA sequencing
SPIRIT: Standard Protocol Items: Recommendations for Interventional Trials
TNF: Tumor necrosis factor
VO_2_: Oxygen consumption
